# Extensive *ERG11* mutations associated with fluconazole-resistant *Candida albicans *isolated from HIV-infected patients

**DOI:** 10.18502/cmm.5.3.1739

**Published:** 2019-09

**Authors:** Sony Paul, Iyanar Kannan, Kalyani Mohanram

**Affiliations:** 1 Department of Microbiology, Tagore Medical College and Hospital, Rathinamangalam, Chennai, India; 2 Department of Microbiology, Saveetha Medical College and Hospital, Chennai, India

**Keywords:** AIDS, Antifungal resistance, Candida species, Candidiasis, ERG11, Fluconazole, Mutation

## Abstract

**Background and Purpose:**

Azoles are preferred antifungal agents given their inexpensiveness, limited toxicity, and potentiality of oral administration. However, the extensive use of prophylactic azole therapy for chronic infections, especially in immunocompromised patients, has led to an increase in azole resistance, thereby rising health care costs. Fluconazole resistance is associated with poor clinical outcomes and the emergence of new infections. The present study aimed to investigate the mutations of *ERG11 *gene in fluconazole-resistant *Candida albicans* isolates.

**Materials and Methods:**

This study was conducted on 80 clinical samples collected from HIV-infected patients with suspected candidiasis in Tagore Medical College Hospital and Government Hospital of Thoracic Medicine, Chennai, India, for a period of 18 months (May 2016-December 2017). The antifungal susceptibility pattern was determined by agar diffusion and broth dilution techniques as per the Clinical and Laboratory Standards Institute guidelines. The *ERG11* gene of the known fluconazole-resistant strains of *C. albicans* was amplified by polymerase chain reaction (PCR). In addition, the samples were subjected to sequencing and mutation analysis.

**Results:**

A total of 60 *Candida* species were isolated from HIV patients and were speciated using standard, conventional, and molecular methods. *Candida albicans* comprised 28.3% (n=17) of the *Candida* isolates, 59% (n=10) of which were resistant to fluconazole. Sequencing of the amplified product of *ERG11* *C. albicans* gene isolates showed that they were highly mutated and included many nonsense mutations which were not reported earlier.

**Conclusion:**

The molecular characterization of *ERG11* gene showed many missense and nonsense mutations. Such mutations, which were unique to the geographical area under investigation, could be concluded to account for the development of resistance to fluconazole.

## Introduction

*Candida *species is a commensal and important opportunistic human pathogen which causes common ailments, such as oral thrush, vaginitis, and invasive infections, in immunocompromised patients. Presently, *Candida *species is the fourth most common cause of nosocomial bloodstream infections (BSIs). Moreover, it is the third leading causative agent of catheter-associated bloodstream infection in the United States with a mortality rate of up to 49% [1-5]. In the European and North American Intensive Care Units, it is the most important fungal infection, ranked after *Staphylococcus aureus* [6]. Furthermore, *Candida* is the first among the top ten bloodstream pathogens despite regional differences [7]. Extensive use of prophylactic azole therapy for chronic infections, especially in patients under long-term antibiotic therapy, as well as steroids or chemotherapy, has resulted in an increase in azole resistance.

Azoles are preferred antifungal agents given their inexpensiveness, limited toxicity, and potentiality for oral administration. Azoles inhibit the target enzyme lanosterol 14α-demethylase, resulting in impaired ergosterol synthesis, thereby disrupting the fungal cell membrane. The *Erg11p*, a member of cytochrome *P450* superfamily, is essential for ergosterol synthesis. It has 528 amino acids with 13 α helices from A to M and several β pleated sheets. The *Erg11p* active center is located deep inside the protein, near the hemochrome between helices I and L. The substrate interacts with a long access channel and is then demethylated [8-12]. Azoles block this process and inhibit ergosterol synthesis*. *The* ERG11* contains 1851 bp, with the transcription start codon being located at 148-150 bp and stop codon at 1732-1734 bp [13]. 

There are multiple mechanisms for azole resistance. The first is based on the changes in the molecular configuration of the target enzyme 14α-demethylase (*Erg11p*) due to mutations in the encoding gene *ERG11*. It can reduce the affinity between azole and protein, often resulting in azole resistance. Point mutation occurrence is associated with an increase in the minimum inhibitory concentration (MIC) of azoles. In addition, the upregulation of *ERG11*, *Candida* drug resistance genes (i.e., *CDR1* and *CDR2*), and multiple drug resistance genes (e.g., *MDR1*) can effectively decrease intracellular drug accumulation. In addition, biofilm is another mediator for antifungal resistance. Sequestration of fluconazole within intracellular vacuoles can be another novel mechanism of resistance. With this background in mind, the present study was conducted to investigate the molecular characterization of *ERG11* gene of certain fluconazole-resistant *C. albicans *and identify mutations that may account for fluconazole resistance.

## Materials and Methods

***Isolates and type strains***

The study was conducted after the approval of Institutional Ethics Committee (IEC24/March 2016) of Tagore Medical College Hospital and Government Hospital of Thoracic Medicine. In line with the ethical principles of research, informed consent was obtained from all participants. A total of 80 samples were included in the study, out of which *Candida* species (n=60) were isolated. The samples included oropharyngeal swabs, nail, sputum, and blood from patients with clinical indicator conditions for HIV. For the purpose of the study, *C. albicans *(MTCC3017), *C. glabrata *(MTCC3019), *C. krusei* (MTCC 9215), and *C. tropicalis *(MTCC3421) were obtained from Microbial Type Culture Collection, Chandigarh, India, to be used as control strains. 

***Media, Drugs, and Reagents***

Sabouraud’s dextrose agar (SDA; HiMedia, India) was prepared according to the manufacturer’s instructions. In this study, CHROM agar (HiMedia, India) was prepared as recommended by the manufacturer. In addition, species identification was accomplished using corn meal agar (CMA; HiMedia, India) with Tween 80, yeast nitrogen base (YNB; HiMedia, India) for carbohydrate assimilation, and liquid medium for sugar fermentation test with 2% carbohydrates solution of dextrose, maltose, sucrose, lactose, maltose, and urease. Mueller Hinton agar (MHA; HiMedia, India) with 2% glucose and methylene blue (0.5 µg/ml) and Roswell Park Memorial Institute (RPMI) 1640 broth (with glutamine and without bicarbonate and with phenol red indicator; Himedia, India) were used for antifungal susceptibility testing. Furthermore, yeast peptone dextrose (YPD; HiMedia, India) broth was utilized for DNA extraction.

In this study, fluconazole (25 µg; Oxoid^TM^, India), voriconazole (1 µg; Oxoid^TM^, India), caspofungin (e-strip; HiMedia EM119, India), fluconazole (e-strip; HiMedia EM072, India), and amphotericin B (Sigma Aldrich) powder were included. In addition, glucose, sucrose, lactose, maltose, trehalose, galactose, and xylose discs supplied from HiMedia were used for assimilation tests. The DNA extraction was performed using HiPuraYeast DNA kit made by Himedia.

***Isolate Identification***

Clinical specimens were inoculated onto SDA slants. The isolates were speciated by CHROM agar (Himedia, India), germ tube formation at 37^°^C, production of chlamydospore in CMA, urease production at 25^°^C, growth at 45^°^C, sugar assimilation, and fermentation tests. Internal transcribed spacer (ITS) 1, 4 sequencings were accomplished for the identification of inconclusive strains using fungal primers for ITS1 (5′- TCCGTAGGTGAACCTGCGG-3′) and ITS4 (5′- TCCTCCGCTTATTGATATGC -3′) (Eurofins India Pvt Ltd), amplifying the ITS region of the ribosomal subunit [14].

***Antifungal Susceptibility Testing***

Susceptibility testing was carried out according to the Clinical Laboratory Standards Institute (CLSI) M44. It is a reference document for antifungal disc diffusion test of yeasts [15]. Sensitivity to fluconazole and voriconazole was evaluated by disc diffusion assays in modified MHA. All the fluconazole-resistant strains were tested for MIC using fluconazole e-strips; additionally, the MICs of caspofungin were also evaluated by e-strips. Susceptibility pattern against amphotericin B was tested by broth dilution method in RPMI 1640 broth, according to the CLSI document M27 A3. In addition, the antifungal susceptibility testing of the yeasts was accomplished using the reference method for broth dilution [16]. In addition, the standard strains of *C. albicans* (MTCC 3017), *C. glabrata *(MTCC 3019), *C. krusei* (MTCC 9215), and *C. tropicalis* (MTCC 3421) were included as quality controls. 

***DNA Extraction***

Fluconazole-resistant *C. albicans* strains were grown on SDA at 37^°^C for 24 h. The strains were inoculated onto YPD broth and incubated for 18-24 h. Total genomic DNA from resistant *C. albicans* isolates was extracted using the HiPure yeast DNA kit according to the manufacturer’s instructions. DNA concentrations and DNA/RNA ratio were measured by BioPhotometer D30 (eppendorf).

***Polymerase chain reaction amplification and gene sequences of ERG11 gene***

The *ERG11* genes were amplified by polymerase chain reaction (PCR; Bio-Rad) using the DNA templates of fluconazole-resistant *C. albicans* which were isolated from primer sequences, namely forward 5’GTT GAA ACT GTC ATT GAT GG 3’ and reverse 5’TCA GAA CAC TGA ATC GAA AG 3’ (Eurofins, India, Pvt Ltd) [13]. For all PCRs, a 25-µL mixture contained 12.5 µl PCR master mix, 2.5 µl genomic DNA, 2.5 µl of each both primer and 5 µl deionized water. The thermocycling conditions for PCR reactions included 30 cycles at 92^°^C for 3 min, 92^°^C for 1 min, 55^°^C for 2 min, and 72^°^C for 1 min, followed by an extension step at 72^°^C for 10 min. 

The PCR product size was analyzed preliminarily by agarose gel electrophoresis (1.5% concentration). Consequently, they were visualized and analyzed in Gel Doc XR+ (Bio Rad). The amplified products of the isolates were purified and sequenced with an ABI 3730 XL DNA analyzer (Applied Biosystems) using standard protocols (Big Dye Terminator v3.1 cycle sequencing Kit-Applied Biosystems) and previously designed primers [14]. For each strain, the entire *ERG11* open reading frame sequence was compared with *ERG11* sequences from the National Center for Biotechnology Information (NCBI).

## Results

***Species identification***

A total of 60 *Candida* species were isolated from 80 clinical specimens. Among the 60 isolates, 16 (26.7%) cases were *C. albicans, *and the remaining 44 (73.3%) isolates were non-*albicans Candida *species. *Candida tropicalis* was identified as the predominant non-*albicans Candida* species (30%). The distribution of *Candida* isolates is presented in Table 1.

***Antifungal susceptibility test***

In this study, 73.3% (n=44) of the *Candida* isolates were resistant to fluconazole. The sensitivity pattern differed among isolates. *Candida glabrata* and *C. tropicalis* showed increased MICs. The antifungal sensitivity patterns of the *Candida* isolates are shown in Table 2. All *C. albicans* isolates showed 100% sensitivity to amphotericin B and caspofungin. The MIC breakpoints of the fluconazole-resistant strains ranged from > 8 to > 256 µg/ml. All the isolates were sensitive to amphotericin B, and only 3 non-*albicans Candida* isolates were resistant to caspofungin. The MICs of amphotericin B and caspofungin had a range of 0.25-1 to 0.012-2 µg/ml, respectively.

Over the last few decades, fungal infections are increasingly alarming, posing great challenges to healthcare professionals. Increase in the number of immunocompromised patients has been significantly contributed to a rise in candidiasis. Due to the variable clinical entities of candidiasis, it is highly important to identify this pathogen in all routine culture specimens received at the laboratory, irrespective of clinician’s suspicion. There are also some reports regarding the emergence of non-*albicans Candida* species as an important pathogen. Hence, the identification of species level, along with antifungal susceptibility, becomes essential as species differ in antifungal susceptibility pattern.

In the present study, 75% of the HIV-seropositive patients suffered from one or other entities of candidiasis. Earlier studies also reported a high incidence of candidiasis in HIV-seropositive patients [17-22]. Although in most studies, *C. albicans* remains the predominant species, in this study, non-*albicans Candida* species outnumbered *C. albicans* in concordance with earlier investigations [23, 24].

***Molecular characterization of ERG11 gene of*** ***Candida albicans***

In the current study, the *ERG11* gene of fluconazole-resistant *C. albicans* was amplified. All the resistant isolates showed mutations in *ERG11* gene, in particular, two sequences exhibited extensive mutations. The *ERG11 *coding region amplified by 

**Table 1:** Distribution of *Candida *isolates among HIV patients

**Species**	**Total isolates**
*C. albicans*	17 (28.3%)
*C. tropicalis*	18 (30%)
*C. krusei*	9 (15%)
*C. parapsilosis*	6 (10%)
*C. glabrata*	5 (8.3%)
Other *Candida* species	5 (8.3%)

**Table 2:** Antifungal susceptibility pattern of *Candida* species

***Candida species***	**Fluconazole**			**Voriconazole**				**Amphotericin B**			**Caspofungin**		
	**S**	**SDD**	**R**	**S**	**SDD**		**R**	**S**	**SDD**	**R**	**S**	**SDD**	**R**
*C. albicans*	1	2	14	10	2		5	16	0	1	15	1	1
*C. tropicalis*	1	5	12	12	3		3	17	1	0	17	0	1
*C. krusei*	0	0	9	5	2		2	8	1	0	8	1	0
*C. glabrata*	3	1	1	5	0		0	5	0	0	5	0	0
*C. kefyr*	0	0	1	1	0		0	1	0	0	1	0	0
*C. parapsilosis*	1	2	3	5			0	6	0	0	6	0	0
*C.dubliniensis*	0	0	1	1	0		0	1	0	0	1	0	0
*C. orthopsilosis*	0	0	1	1	0		0	1	0	0	1	0	0
*K. ohmeri*	0	0	1	0	0		1	1	0	0	1	0	0
*H. opuntiae*	0	0	1	1	0		0	-	-	-	1	0	0
**Combined Data**													
*C. albicans*	5	2	10	10		2	5	16	0	1	15	1	1
Non-*albicans Candida* species	5	8	30	31		6	6	40	2	0	41	1	1
*P*	>0.05			>0.05				>0.05			>0.05		

**Figure 1:**
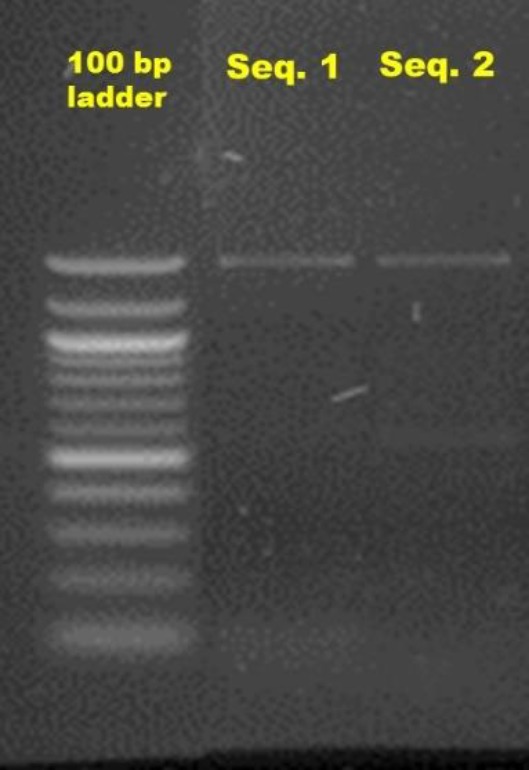
Agarose gel electrophoresis of polymerase chain reaction amplified products of *ERG11* gene

**Figure 2:**
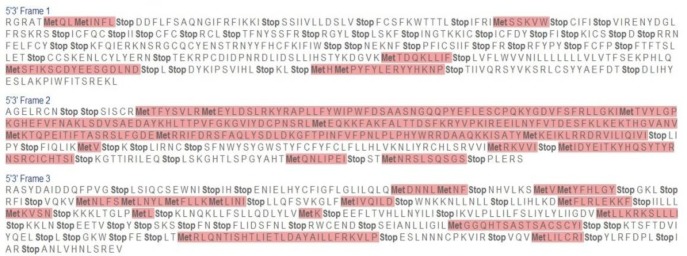
Translated sequences in three different frames in Expasy Translate tool of sequence 1 showing stop codons

**Figure 3:**
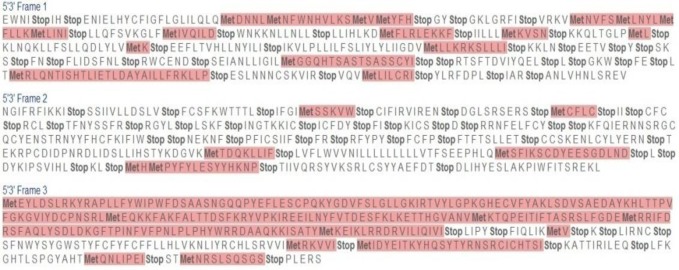
Translated sequences in three different frames in Expasy Translate tool of sequence 2 showing stop codons

The *ERG11* gene sequences were subjected to multiple sequence alignment (MSA) using the online server tool TCoffee with the wild type gene sequence GenBank accession number AF153844. The MSA of sequences 1 and 2 showed that they were highly mutated by possessing all types of mutations. Moreover, in all three translation frames, it possessed many stop codons (i.e., nonsense mutation). Frames 1, 2, and 3 of sequence 1 showed 37, 12 and 34 stop codons, respectively (Figure 2). Therefore, it is evident that gene can produce only a truncated polypeptide chain, thereby resulting in aberrant protein. The MSA of sequence 2 revealed that it also possessed many stop codons. In frames 1, 2, and 3, it showed 32, 36, and 10 stop codons, respectively (Figure 3). Hence, this gene-translated product also may result in a truncated protein.

The proper functional coding sequence of the *ERG11* gene of both strains was prepared by trimming and translated to protein sequence in Expasy online translate server tool. These two protein sequences were subjected to BLASTP. They showed similarity with lanosterol 14α-demethylase, the gene product of *ERG11* with 94% identity (E-value=1e^-156^) and 93% (E-value=2e^-154^) for sequences 1 and 2, respectively. The missense mutations of both translated products were also analyzed. Table 3 shows various missense mutations present in the translated products of both sequences.

**Table 3:** Missense mutations of *Candida albicans* in the translated product of *ERG11* sequences 1 and 2

**Seq1**	Y18D, D23Y, V28L, G36D, Y41N, F49L, R53P, M63R, E243D, 245-283 missense mutations
**Seq2**	Y3D, D8Y, V13L, G21D, Y26N, F34L, R38P, F47L, M48G, M54R, F65C, N68Y, K142T, E229D

***Nucleotide Sequence Accession Number***

In this study, the *ERG11* gene sequences of two *C. albicans* clinical isolates were deposited in the GenBank database under the accession numbers of MF155016 and MF175064.

## Discussion

The purpose of the molecular characterization of *ERG11* gene in the present study was to understand the molecular mechanism of fluconazole resistance in *C. albicans* isolated from two HIV-infected patients. The sequences of the *ERG11* gene of two *C. albicans* species in the present study showed that they are highly mutated and possess all types of mutations; therefore, the mutation may be responsible for the development of fluconazole resistance. Many similar investigations addressing *ERG11* gene mutation reported different types of point mutations [12, 25]. However, these studies showed only a limited number of mutations, especially missense mutations.

However, in the present study, the *ERG11 *gene of both strains of *C. albicans* demonstrated many nonsense mutations, along with a huge number of missense mutations. Hence, it is clear that the *ERG11* of these two strains produces a defective enzyme and contributes to fluconazole resistance property [13, 17]. The sterol 14 alpha-demethylase enzyme produced by mutated *ERG11* gene has an altered conformation which makes the enzyme to lose its ability to bind to azoles [26]. It can also be considered as a screening tool to identify resistant strains.

## Conclusion

The molecular characterization of *ERG11* gene showed an extensive mutation that may contribute to the resistance of *C. albicans* to fluconazole. Furthermore, the high prevalence of fluconazole-resistant strains of *C. albicans* in this particular geographical area in HIV patients indicating the presence of *ERG11 *mutated strains.

## References

[1] Centres for Disease Control and Prevention (US) (2013). Antibiotic resistance threats in the United States, 2013. New York: Centres for Disease Control and Prevention.

[2] Edmond MB, Wallace SE, McClish DK, Pfaller MA, Jones RN, Wenzel RP (1999). Nosocomial bloodstream infections in United States hospitals: a three-year analysis. Clin Infect Dis.

[3] Gudlaugsson O, Gillespie S, Lee K, Vande Berg J, Hu J, Messer S (2003). Attributable mortality of nosocomial candidemia, revisited. Clin Infect Dis.

[4] Hidron AI, Edwards JR, Patel J, Horan TC, Sievert DM, Pollock DA (2008). NHSN annual update: antimicrobial-resistant pathogens associated with healthcare-associated infections: annual summary of data reported to the National Healthcare Safety Network at the centres for Disease Control and Prevention, 2006-2007. Infect Control Hosp Epidemiol.

[5] Vincent JL, Rello J, Marshall J, Silva E, Anzueto A, Martin CD (2009). International study of the prevalence and outcomes of infection in intensive care units. JAMA.

[6] Davoudi AR, Najafi N, Hoseini Shirazi M, Ahangarkani F (2014). Frequency of bacterial agents isolated from patients with nosocomial infection in teaching hospitals of Mazandaran University of Medical Sciences in 2012. Caspian J Intern Med.

[7] Eggimann P, Bille J, Marchetti O (2011). Diagnosis of invasive candidiasis in the ICU. Ann Intensive Care..

[8] Ji H, Zhang W, Zhou Y, Zhang M, Zhu J, Song Y (2000). A three dimensional model of lanosterol 14alpha-demethylase of Candida albicans and its interaction with azole antifungals. J Med Chem.

[9] Marichal P, Koymans L, Willemsens S, Bellens D, Verhasselt P, Luyten W (1999). Contribution of mutations in the cytochrome P450 14α-demethylase (Erg11p, Cyp51p) to azole resistance in Candida albicans. Microbiology.

[10] Podust LM, Poulos TL, Waterman MR (2001). Crystal structure of cytochrome P450 14a -sterol demethylase (CYP51) from Mycobacterium tuberculosis in complex with azole inhibitors. Proc Natl Acad Sci U S A..

[11] Sanglard D, Ischer F, Koymans L, Bille J (1998). Amino acid substitutions in the cytochrome P-450 lanosterol 14 a-demethylase (CYP51A1) from azole-resistant Candida albicans clinical isolates contribute to resistance to azole antifungal agents. Antimicrob Agents Chemother.

[12] Nabili M, Abdollahi Gohar A, Badali H, Mohammadi R, Moazeni M (2016). Amino acid substitutions in Erg11p of azole-resistant Candida glabrata: possible effective substitutions and homology modelling. J Glob Antimicrob Resist..

[13] Xu Y, Chen L, Li C (2008). Suceptibility of clinical isolates of Candida species to fluconazole and detection of Candida albicans ERG11 mutations. J Antimicrob Chemother.

[14] Kathuria S, Singh PK, Sharma C, Prakash A, Masih A, Kumar A (2015). Multidrug-resistant Candida auris misidentified as Candida haemulonii: characterization by matrix-assisted laser desorption ionization–time of flight mass spectrometry and DNA sequencing and its antifungal susceptibility profile variability by Vitek 2, CLSI broth microdilution, and E test method. J Clin Microbiol.

[15] CLSI (2009). Method for Antifungal disc diffusion Susceptibility testing of yeasts.

[16] CLSI (2008). Reference method for broth dilution antifungal susceptibility testing of yeasts; approved standard.

[17] Perea S, Lopez-Ribot JL, Kirkpatrick WR, McAtee RK, Santillán RA, Martínez M (2001). Prevalence of molecular mechanisms of resistance to azole antifungal agents in Candida albicans strains displaying high level fluconazole resistance isolated from human immunodeficiency virus infected patients. Antimicrob Agents Chemother.

[18] Wadhwa A, Kaur R, Agarwal SK, Jain S, Bhalla P (2007). AIDS-related opportunistic mycoses seen in a tertiary care hospital in North India. J Med Microbiol.

[19] Nagalingeswaran K, Solomon S, Madhivanan P, Yepthomi T, Venkatesan C, Amalraj E (2000). Correlation between plasma viral load and CD4+T cell count to opportunistic infections in persons with HIV in South India. Int Conf AIDS..

[20] Pruthvi BC, Vikram S, Suman SK, Jayaprakash B, Rau NR (2008). Spectrum of clinical presentation and opportunistic infections in HIV: an Indian scenario. Int J Infect Dis..

[21] Pandey S, Sundar S, Hasan H, Shankar R, Singh RP (2008). Clinical profile and opportunistic infection in HIV/AIDS patients attending SS Hospital, Varanasi. Indian J Prev Soc Med.

[22] Singh A, Bairy I, Shivananda PG (2003). Spectrum of opportunistic infections in AIDS cases. Indian J Med Sci.

[23] Deorukhkar S, Saini S, Stephen M (2014). Non-albicans Candida infections: an emerging threat. Interdiscip Perspect Infect Dis..

[24] Nguyen MH, Peacock JE Jr, Morris AJ, Tanner DC, Nguyen ML, Snydman DR (1996). The changing face of candidemia: emergence of non-Candida albicans species and antifungal resistance. Am J Med.

[25] Goldman GH, da Silva Ferreira ME, dos Reis Marques E, Savoldi M, Perlin D, Park S (2004). Evaluation of fluconazole resistance mechanisms in Candida albicans clinical isolates from HIV-infected patients in Brazil. Diagn Microbiol Infect Dis.

[26] Lamb DC, Kelly DE, Schunck WH, Shyadehi AZ, Akhtar M, Lowe DJ (1997). The mutation T315A in Candida albicans sterol 14alpha-demethylase causes reduced enzyme activity and fluconazole resistance through reduced affinity. J Biol Chem.

